# Supplementary material for: Alpha-synuclein is strategically positioned for afferent modulation of midbrain dopamine neurons and is essential for cocaine preference
Source: Commun Biol. 2019 Nov 15;2:418. doi: 10.1038/s42003-019-0651-8 (PMC6858354; doi:10.1038/s42003-019-0651-8)
Supplement: Supplementary file 1 — Supplementary Information [file 42003_2019_651_MOESM1_ESM.pdf]

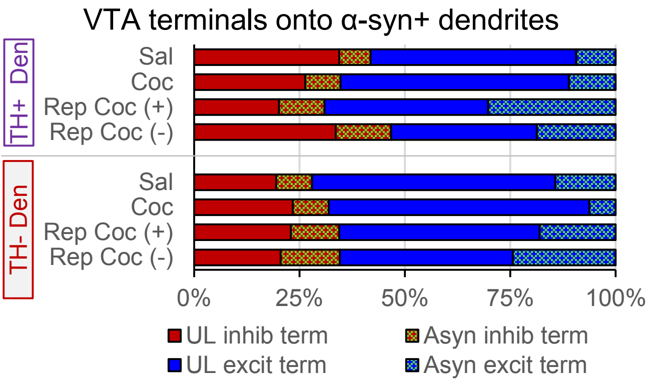

Supplementary Figure 1: Quantification of characterized synaptic contacts onto TH+ and TH- dendrites that also contain α-syn labeling in the VTA. When systemically present, repeated cocaine (rep coc +) increased α-syn immunolabeling in axon terminals making excitatory type synaptic contacts onto dopamine dendrites (TH+), a finding similar to dendrites without α-syn labeling (characterized in Fig 4B), but to a lesser extent.

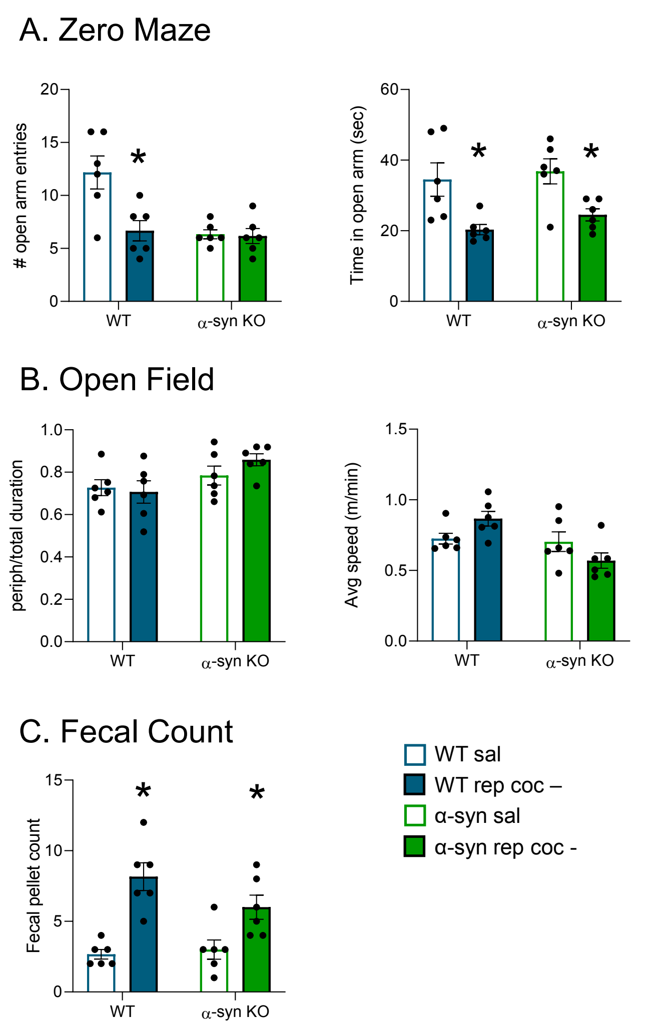

Supplementary Figure 2: Bar graphs showing increased anxiety-like behaviors 72 hours after repeated cocaine administration. A) WT and α-syn KO mice show decreased entries and time spent in the open areas of the zero maze. B) There were no differences in the ratio of time spent in the periphery vs. the center of the open field (left) or ambulatory speed (right) by cocaine exposure or genotype. C) Cocaine exposure in both WT and α-syn KO increased the rate of defecation in a 30 minute time period. * = $p < 0.05$.

Original blot images for Fig. 3A

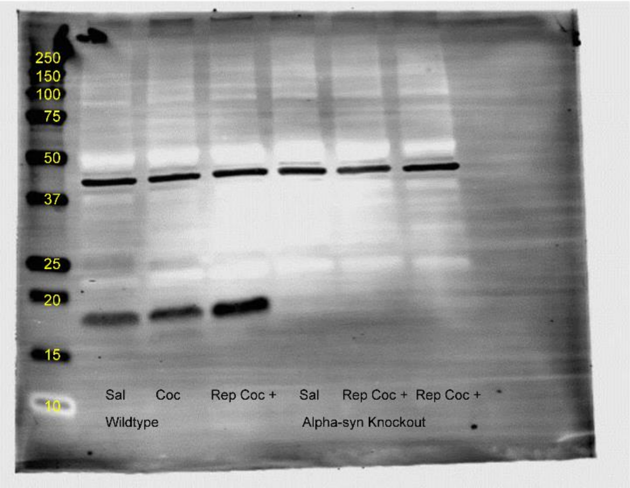

Original blot images for Fig. 6A

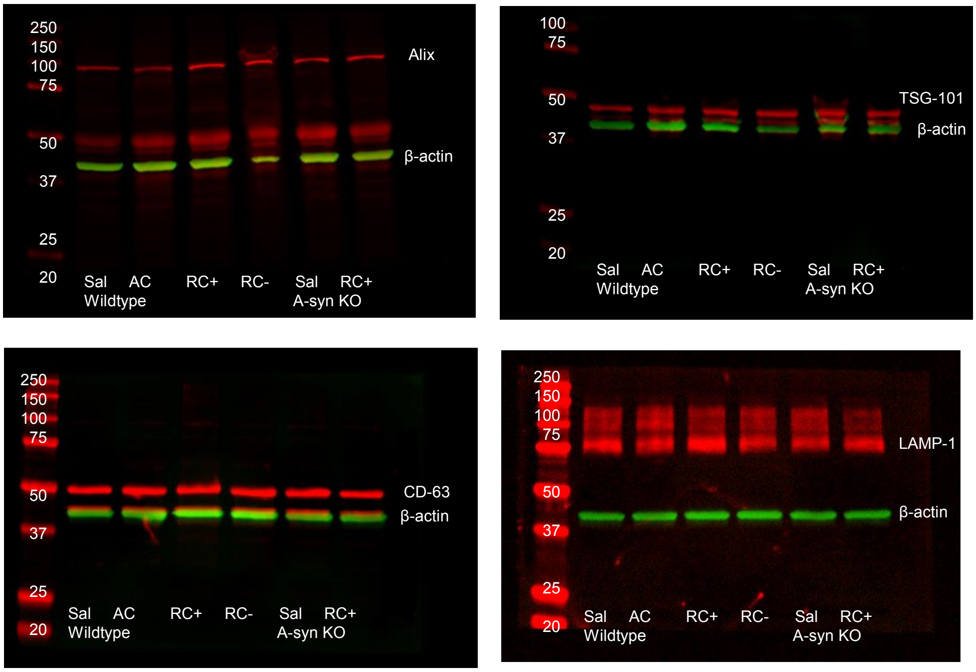

Original blot images for Fig. 6C

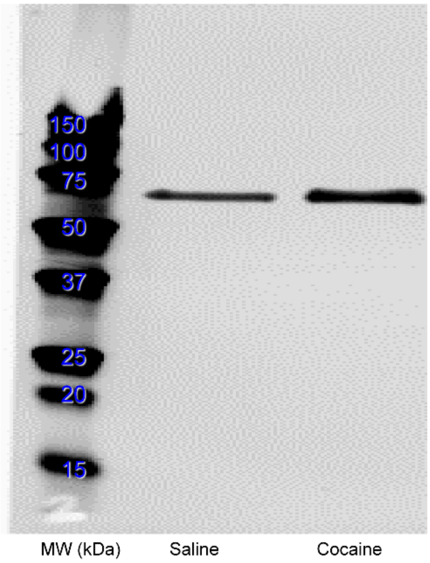

Supplementary Figure 3: Original blot images corresponding to those shown in the main figures.

Supplementary Table 1: 72-hour somatic withdrawal symptom assessment.

	Wildtype Mice:						Alpha-synuclein Knockout Mice:					
	M1	M2	M3	M4	M5	M6	M1	M2	M3	M4	M5	M6
Teeth Chattering		X								X		
Salivation												
Shaking							X	X			X	
Ptosis												
Tachypnea												
Urination	X	X	X	X	X	X	X	X	X	X	X	X
Defecation	X	X	X	X	X	X	X	X	X	X	X	X
Diarrhea												
Penile discharge												
Wide stance												
Self-stimulation												
Escape Attempts			X									
Vocalization												
Exophthalmoses												
Tearing												
Writhing												
Symptom severity	1	1	1	1	1	1	1	2	1	2	1	1

Symptom severity: 1=mild; 10=extreme
