# Supplementary material for: Alpha-synuclein is strategically positioned for afferent modulation of midbrain dopamine neurons and is essential for cocaine preference
Source: Commun Biol. 2019 Nov 15;2:418. doi: 10.1038/s42003-019-0651-8 (PMC6858354; doi:10.1038/s42003-019-0651-8)
Supplement: Supplementary file 2 — Description of Additional Supplementary Files [file 42003_2019_651_MOESM2_ESM.docx]

Description of Additional Supplementary Files

Supplementary Data 1.xls: Source data underlying plots.
