# Supplementary material for: Alpha-synuclein is strategically positioned for afferent modulation of midbrain dopamine neurons and is essential for cocaine preference
Source: Commun Biol. 2019 Nov 15;2:418. doi: 10.1038/s42003-019-0651-8 (PMC6858354; doi:10.1038/s42003-019-0651-8)
Supplement: Supplementary file 4 — Reporting Summary [file 42003_2019_651_MOESM4_ESM.pdf]

nature research

Corresponding author(s): Diane A. Lane
Last updated by author(s): Sep 9, 2019

Reporting Summary

Nature Research wishes to improve the reproducibility of the work that we publish. This form provides structure for consistency and transparency in reporting. For further information on Nature Research policies, see Authors & Referees and the Editorial Policy Checklist.

Statistics

For all statistical analyses, confirm that the following items are present in the figure legend, table legend, main text, or Methods section.

n/a	Confirmed	
☐	☒	The exact sample size (n) for each experimental group/condition, given as a discrete number and unit of measurement
☐	☒	A statement on whether measurements were taken from distinct samples or whether the same sample was measured repeatedly
☐	☒	The statistical test(s) used AND whether they are one- or two-sided<br>*Only common tests should be described solely by name; describe more complex techniques in the Methods section.*
☐	☒	A description of all covariates tested
☐	☒	A description of any assumptions or corrections, such as tests of normality and adjustment for multiple comparisons
☐	☒	A full description of the statistical parameters including central tendency (e.g. means) or other basic estimates (e.g. regression coefficient) AND variation (e.g. standard deviation) or associated estimates of uncertainty (e.g. confidence intervals)
☐	☒	For null hypothesis testing, the test statistic (e.g. F, t, r) with confidence intervals, effect sizes, degrees of freedom and P value noted<br>*Give P values as exact values whenever suitable.*
☐	☒	For Bayesian analysis, information on the choice of priors and Markov chain Monte Carlo settings
☐	☒	For hierarchical and complex designs, identification of the appropriate level for tests and full reporting of outcomes
☐	☒	Estimates of effect sizes (e.g. Cohen's d, Pearson's r), indicating how they were calculated

Our web collection on statistics for biologists contains articles on many of the points above.

Software and code

Policy information about availability of computer code

Data collection	Data was collected using Stoelting Any-Maze (ver. 4.99), Med-Associates Activity Monitor software (ver. 7.06), Advanced Microscopy CCD camera system (ver. 3.1).
Data analysis	Data analysis was conducted using Leica Image Studio (ver. 5.2), Microcomputer Image Device software (MCID, ver 7.0), Microsoft Excel (Excel 2013). NIH Image J software, and IBM SPSS statistics (Version 20).

For manuscripts utilizing custom algorithms or software that are central to the research but not yet described in published literature, software must be made available to editors/reviewers. We strongly encourage code deposition in a community repository (e.g. GitHub). See the Nature Research guidelines for submitting code & software for further information.

Data

Policy information about availability of data

All manuscripts must include a data availability statement. This statement should provide the following information, where applicable:
- Accession codes, unique identifiers, or web links for publicly available datasets
- A list of figures that have associated raw data
- A description of any restrictions on data availability

All data is readily available in a file containing figures and raw data. Public datasets were not used and there is no restriction to data availability.

Field-specific reporting

Please select the one below that is the best fit for your research. If you are not sure, read the appropriate sections before making your selection.

☐ Life sciences ☒ Behavioural & social sciences ☐ Ecological, evolutionary & environmental sciences

Behavioural & social sciences study design

All studies must disclose on these points even when the disclosure is negative.

Study description	The study utilized behavioral, microscopic, and immunoblot techniques and most variables were quantitative, although some descriptive and proportional qualitative data were also included.
Research sample	Behavioral, molecular, and microscopic data were obtained from wildtype (C57BL6/J) and alpha-synuclein knockout (C57BL/6N-Sncatm1Mjff/J) mice.
Sampling strategy	Mice were randomly assigned to experimental conditions and the number of mice and number of neuronal profiles utilized for analysis was determined by power analysis of prior similar studies. In addition, power analysis and R-squared was evaluated on collected data to ensure there was enough statistical power for the reported experimental findings. For qualitative data, collection of data points was discontinued once data saturation was reached.
Data collection	Behavioral data<br>Cocaine injections were completed immediately prior to behavioral measures, so the researcher was not completely blind to experimental groups during data collection, however, wildtype and alpha-synuclein knockout mice were number coded and unknown during data collection. Conditioned place preference and locomotor activity was automatically collected within appropriate test chambers using Activity Monitory software (Med-Associates) and exported directly into Excel for data analysis. Barnes Maze data was collected via video into ANY-Maze software that automatically assessed latency and errors that were directly exported to Excel for data analysis. Sweetened condensed milk and water intake was measured daily by weight by the researcher who was blind to mouse genotype and entered manually into Excel.<br>Immunoblot data<br>Samples were provided to researchers that were blind to experimental treatment for Western Blot immunolabeling and analyzed using Leica Image Studio software for photometric analysis of immunoblot intensities that were directly exported into Excel for analysis.<br>Microscopy<br>All tissue was punched for identification and processed together for immunocytochemistry to prevent any bias from different immuno runs. After immunolabeling, the tissue was number coded and subsequent imaging and processing was done blind by the researcher. MCID and Image J morphometric software was used to calculate size, density, and labeling density of immuoreagents. The numeric code was used during all data collection procedures and only revealed for finals statistical analysis.<br>Exosome Antibody Array Immunoblot<br>The blot papers were number coded and evaluated using Leica Image Studio software for photometric analysis of immunoblot intensities by a researcher blind to the experimental conditions and directly exported into Excel for analysis.
Timing	CD63 immunolabeling and electron microscopic analysis 3/18/16 – 5/23/16; additional studies 6/15/19-8/10/19<br>CPP and locomotor behavior analysis 5/17/16 – 6/28/17; additional studies 6/15/19-8/10/19<br>Exosome Isolation and antibody array studies 9/21/16 – 10/30/16<br>Western Blot analyses 6/19/18 – 7/8/18<br>Barnes Maze testing and analysis 11/13/17 – 11/28/17<br>Electron microscopic immunolabeling and analysis 5/20/16 – 12/8/17; additional studies 6/15/19 - 8/10/19<br>Sweetened condensed milk intake and CPP behavioral testing 4/23/18 – 5/21/18
Data exclusions	No data was excluded from the raw datasets.
Non-participation	No participants dropped out of the studies presented in our manuscript.
Randomization	Mice were randomly assigned to experimental groups by assigning numbers written on paper that was blindly pulled from a small container.

Reporting for specific materials, systems and methods

We require information from authors about some types of materials, experimental systems and methods used in many studies. Here, indicate whether each material, system or method listed is relevant to your study. If you are not sure if a list item applies to your research, read the appropriate section before selecting a response.

Materials & experimental systems

n/a	Involved in the study	
☐	☒	Antibodies
☒	☐	Eukaryotic cell lines
☒	☐	Palaeontology
☐	☒	Animals and other organisms
☒	☐	Human research participants
☒	☐	Clinical data

Methods

n/a	Involved in the study	
☒	☐	ChIP-seq
☒	☐	Flow cytometry
☒	☐	MRI-based neuroimaging

Antibodies

Antibodies used

Primary antibodies used:
1. α-syn (1:2000 dilution, BD Biosciences, #610787)
2. CD63 (1:2000 dilution, Millipore Sigma, #SAB4301607)
3. ALIX (1:2000 dilution, ThermoFisher Scientific, #MA1-83977)
4. LAMP-1 (1:1000 dilution, ABCAM, #AB208943)
5. TSG-101 (1:2000 dilution, ThermoFisher Scientific, #MA1-23296)
6. β-actin (1:3000 dilution, Cell Signaling Technology, #3700 or #4970
7. Glutamate (1:500 dilution, Millipore Sigma, AB133)
8. GABA (1:500 dilution, ThermoFisher Scientific, AB175)
9. Tyrosine hydroxylase (1:10,000 dilution, Millipore Sigma, AB1542)

Secondary fluorescent antibodies used:
1. Donkey anti-rabbit Cy3 (1:400 dilution, Jackson Laboratories, #711-165-152)
2. Donkey anti-rat Alexafluor 647 (1:400 dilution, Jackson Laboratories, #712-605-153)
3. Donkey anti-mouse FITC (1:400 dilution, Jackson Laboratories, #715-095-150)

Secondary peroxidase antibodies used:
1. Biotinylated horse-anti-mouse IgG (1:400 dilution, Vector Labs; BA-2001)
2. Biotinylated donkey-anti-rabbit IgG (1:400 dilution, Jackson Laboratories; #711-065-152)

Secondary colloidal gold antibody used;
1. Donkey-anti-sheep colloidal gold (1 nm) IgG (1:50 dilution, EMS, #25820)

Validation

All primary antibodies were validated by omission of the primary antibody during the immunolabeling procedure and for alpha-synuclein, inclusion of knockout tissue. All utilized antibodies showed no labeling with primary antibody exclusion
CD63 - We showed no alpha-synuclein bands on our Western Blots using the antibody against alpha-synuclein.
For all other antibodies, in addition to omission of the primary antibody, specific information was obtained from the manufacturer:
ALIX - Western blot analysis of HeLa whole cell lysate showed specificity of antibody. Certificate of analysis shows specificity in human and mouse tissue.
LAMP-1 – antibody database links: Entrez Gene: 16783 mouse, Swiss Prot: P11438 mouse, Unigene: 16716 mouse, Unigene: 475822 mouse.
TSG-101 – antibody target was verified by knockdown to ensure antibody binds to the antigen stated.
β-actin - Species reactivity: human, mouse, rat, hamster, monkey, dog. Antibody database links: Entrez-Gene ID: 60; Swiss-Prot Acc: P60709.
Glutamate – No cross activity to D-gluatamate, Aspartate, or GABA.
GABA - Recognizes GABA. Staining was blocked by preabsorbing with 100uM GABA conjugated to glutaraldehyde. 500uM of similar conjugations of glutamic acid, glutamate and taurine failed to block staining.
Tyrosine hydroxylase - The antibody gives specific labeling of noradrenergic axons in primate cerebral cortex (Brain Res., 1989, 500:313-324.), reactive in mammals, rat, and mouse. Antibody database: Entrez-Gene ID: 351.

Animals and other organisms

Policy information about studies involving animals; ARRIVE guidelines recommended for reporting animal research

Laboratory animals

Adult (14+ weeks) male wildtype (WT; C57BL66/J) and alpha-synuclein knockout (α-syn KO; C57BL/6N-Sncatm1Mjff/J) mice were utilized for experimental procedures (Jackson Labs, Bar Harbor, ME)

Wild animals

The study did not involved wild animals.

Field-collected samples

The study did not involves samples collected from the field.

Ethics oversight

Experimental protocols were approved by the Institutional Animal Care and Use (IACUC) committee at Weill Cornell Medical College and performed in accordance with the Guidelines for the Care and Use of Laboratory Animals of the National Institutes of Health

Note that full information on the approval of the study protocol must also be provided in the manuscript.
